# Ultra-High Resolution Optical Coherence Tomography Imaging of Unilateral Drusen in a 31 Year Old Woman

**DOI:** 10.23937/2378-3656/1410063

**Published:** 2015-10-10

**Authors:** Talisa E de Carlo, Mehreen Adhi, Chen D Lu, Jay S Duker, James G Fujimoto, Nadia K Waheed

**Affiliations:** 1 New England Eye Center and Tufts Medical Center, Tufts University, Boston, MA, USA; 2 Department of Electrical Engineering and Computer Science, and Research Laboratory of Electronics, Massachusetts Institute of Technology, Cambridge, USA

**Keywords:** Drusen, Optical Coherence Tomography, Retinal Imaging, Retina

## Abstract

We report a case of widespread unilateral drusen in a healthy 31 year old Caucasian woman using multi-modal imaging including ultra-high resolution optical coherence tomography (UHR-OCT). Dilated fundus exam showed multiple drusen-like lesions in the posterior pole without heme or fluid. Fundus auto fluorescence demonstrated hyperautofluorescent at the deposits. Fluorescein angiography revealed mild hyperfluorescence and staining of the lesions. Spectral-domain optical coherence tomography (SD-OCT) OS showed accumulations in the temporal macula at Bruch’s membrane. UHR-OCT provided improved axial resolution compared to the standard 5 μm on the commercial SD-OCT and confirmed the presence of deposits in Bruch’s membrane, consistent with drusen. The retinal layers were draped over the excrescences but did not show any disruption.

## Introduction

Drusen was first described by Dr. Franciscus Donders in 1855 via histopathological observations as round accumulations under the retinal pigment epithelium (RPE) in Bruch’s membrane [1]. They begin as small (< 63 μm) well-circumscribed accumulations (hard drusen) but over time may coalesce into larger, less-defined elevations (soft drusen). While a few drusen may occur in normal eyes, they are infrequently seen in patients under age 55 especially in large numbers [2]. In the Beaver Dam Offspring Study, soft drusen were seen in only 2% of patients age 21–34 [3]. When drusen occur in young adults, they are typically bilateral. A study of 488 Caucasians age 18–54 determined that only 2% of subjects had unilateral drusen and none of these eyes had more than 20 drusen [4].

We report a case of widespread unilateral drusen in a healthy 31 year old Caucasian woman.

## Case Report

A 31 year old Caucasian woman with a history of prematurity was referred to New England Eye Center for drusen-like deposits in the left eye (OS). She denied smoking, renal disease, or a family history of retinal disorders.

**Figure 1:**
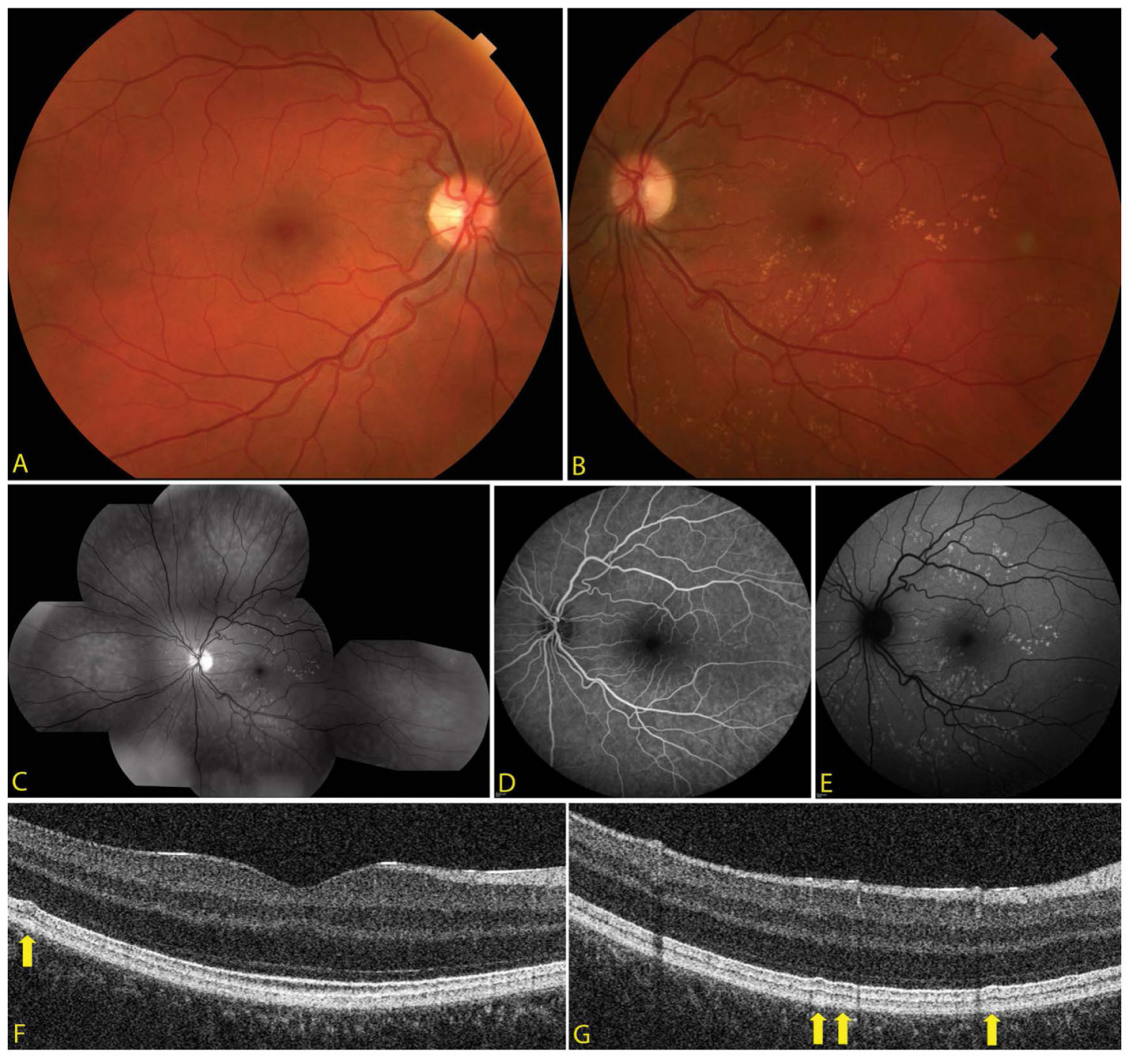
(a) Color fundus photo OD showing no drusen-like deposits in the posterior pole. (b) Color fundus photo OS depicting multiple scattered small drusen-like deposits in the posterior pole without heme or fluid OS. (c) Red-free fundus photo composition OS demonstrating multiple scattered drusen-like deposits centrally. (d) Fluorescein angiography OS revealing mild hyperfluorescence and staining of the deposits but no dark choroid. (e) Fundus autofluorescence OS demonstrating hyperautofluorescence in the areas of the deposits. (f and g) Ultra-high resolution optical coherence tomography b-scans examples of the drusen-like deposits, which are sub-RPE (arrows).

The patient’s uncorrected visual acuity was 20/20 in the right eye (OD) and 20/20-1 OS. Dilated fundus exam showed a normal fundus OD and multiple drusen-like lesions in the posterior pole without heme or fluid OS. Fundus Autofluorescence (FAF) OS demonstrated hyperautofluorescence at the deposits. Fluoresce in Angiography (FA) OS revealed mild hyperfluorescence and staining of the lesions (Figure 1). Spectral-domain optical coherence tomography (SD-OCT) OS showed accumulations in the temporal macula at Bruch’s membrane.

A prototype ultra-high resolution SD-OCT (UHR-OCT) system was employed to provide improved axial resolution of 2–3 μm compared to the standard 5 μm on the commercial SD-OCT [5,6]. The higher resolution provided by the UHR-OCT confirmed the presence of deposits in Bruch’s membrane OS, consistent with drusen. The retinal layers were draped over the excrescences but did not show any disruption (Figure 1).

## Discussion

This report describes a case of unilateral drusen in a healthy 31 year old non-smoking Caucasian woman with no family history of retinal pathology. It is unusual to have extensive macular and extra macular unilateral drusen in young patients. To our knowledge this is the only reported case of widespread unilateral drusen in a patient in her 30s.

The patient had no family history of retinal diseases to suggest an inherited disorder, and lacked the characteristic radial pattern of malattia leventinese [7]. Although she was born premature, her normal vasculature and unilateral nature were inconsistent with retinopathy of prematurity [8]. The deposits were unlikely polar bear tracks as those lesions are typically peripheral, at the IS/OS junction, and hypoautofluroescent [9]. The patient’s exam was most indicative of drusen despite the fact that she lacked any major risk factors for drusen: smoking, elevated HDL, male gender and advanced age [3]. The present case is therefore intriguing in its uniqueness because a young patient without a history of known major risk factors for drusen presented with florid unilateral drusen.
